# Super-Enhancer Dysregulation in Rhabdoid Tumor Cells Is Regulated by the SWI/SNF ATPase BRG1

**DOI:** 10.3390/cancers16050916

**Published:** 2024-02-24

**Authors:** Cheyenne A. Jones, Jing Wang, James R. Evans, Hannah R. Sisk, Carl B. Womack, Qi Liu, William P. Tansey, April M. Weissmiller

**Affiliations:** 1 Department of Biology, Middle Tennessee State University, Murfreesboro, TN 32132, USA; cheyenne.jones@duke.edu (C.A.J.); jre4i@mtmail.mtsu.edu (J.R.E.); hrs4i@mtmail.mtsu.edu (H.R.S.); cbw4d@mtmail.mtsu.edu (C.B.W.); 2 Center for Quantitative Sciences, Vanderbilt University Medical Center, Nashville, TN 37232, USA; jing.wang.1@vumc.org (J.W.); qi.liu@vumc.org (Q.L.); 3 Department of Biostatistics, Vanderbilt University Medical Center, Nashville, TN 37203, USA; 4 Department of Cell and Developmental Biology, Vanderbilt University School of Medicine, Nashville, TN 37240, USA; william.p.tansey@vanderbilt.edu; 5 Department of Biochemistry, Vanderbilt University School of Medicine, Nashville, TN 37240, USA

**Keywords:** BRG1, SWI/SNF, AP-1, super-enhancer, rhabdoid tumor, SMARCA4, SMARCB1

## Abstract

**Simple Summary:**

Rhabdoid tumors are a group of rare and aggressive pediatric cancers that are associated with dismal survival rates. These tumors are classified within the ~20% of all cancers showing mutations in the subunits of the SWI/SNF chromatin remodeling complex. Chromatin remodelers are large protein complexes that control gene expression at least in part through regulating DNA accessibility. In rhabdoid tumor cell lines, which harbor inactivating mutations in the *SMARCB1* gene, the SWI/SNF subunit BRG1—a synthetic lethal target in these cells—retains functionality in chromatin remodeling and regulation of the rhabdoid genome. The aim of this study was to assess the contribution of BRG1 to rhabdoid gene regulation across multiple rhabdoid cell lines. These data provide valuable insights in understanding how gene regulation following *SMARCB1* loss is achieved, which may be broadly applicable to other cancers marked by SWI/SNF subunit mutation.

**Abstract:**

Mutations in the SWI/SNF chromatin remodeling complex occur in ~20% of cancers. In rhabdoid tumors defined by loss of the SWI/SNF subunit *SMARCB1*, dysregulation of enhancer-mediated gene expression is pivotal in driving oncogenesis. Enhancer dysregulation in this setting is tied to retention of the SWI/SNF ATPase BRG1—which becomes essential in the absence of *SMARCB1*—but precisely how BRG1 contributes to this process remains unknown. To characterize how BRG1 participates in chromatin remodeling and gene expression in *SMARCB1*-deficient cells, we performed a genome-wide characterization of the impact of BRG1 depletion in multiple rhabdoid tumor cell lines. We find that although BRG1-regulated open chromatin sites are distinct at the locus level, the biological characteristics of the loci are very similar, converging on a set of thematically related genes and pointing to the involvement of the AP-1 transcription factor. The open chromatin sites regulated by BRG1 colocalize with histone-marked enhancers and intriguingly include almost all super-enhancers, revealing that BRG1 plays a critical role in maintaining super-enhancer function in this setting. These studies can explain the essentiality of BRG1 to rhabdoid tumor cell identity and survival and implicate the involvement of AP-1 as a critical downstream effector of rhabdoid tumor cell transcriptional programs.

## 1. Introduction

The SWI/SNF chromatin remodelers are large multi-subunit complexes that alter histone–DNA contacts within nucleosomes to control the accessibility of DNA [1]. All characterized mammalian SWI/SNF complexes contain a mutually exclusive ATPase subunit, BRG1 or BRM (also called SMARCA4 or SMARCA2, respectively), that uses energy from ATP hydrolysis to drive chromatin remodeling. In addition to an ATPase, SWI/SNF complexes can contain more than 10 additional subunits and have been categorized into three major types of complexes known as canonical BAF (cBAF), non-canonical BAF (ncBAF), and polybromo-associated BAF (pBAF). Each type of complex is defined by the presence of unique SWI/SNF subunits such as DPF2 and ARID1A/B in cBAF; BRD9 and GLTSCR1/1L in ncBAF; and ARID2, PBRM, and BRD7 in pBAF. All three mammalian SWI/SNF complexes localize for the most part to distinct regions of the genome and work together to regulate complex cellular gene expression programs [2]. Mutations in the genes that encode SWI/SNF subunits collectively occur in ~20% of all cancers and are often deleterious to function or block SWI/SNF subunit expression [3,4]. These observations, together with those showing that inactivation of specific SWI/SNF subunits in mouse models results in cancer, has bolstered the idea that SWI/SNF acts as a potent tumor suppressor [5,6,7,8].

Unraveling the ways in which deleterious mutations of SWI/SNF subunits promote and sustain malignancy is an active area of research. Studies in the context of malignant rhabdoid tumor cell lines have been instrumental in advancing our understanding of the molecular events that allow cancer progression upon SWI/SNF subunit loss. Malignant rhabdoid tumors and atypical teratoid rhabdoid tumors (collectively referred to as “rhabdoid tumors” herein) are rare and aggressive pediatric cancers with limited treatment options [9,10]. To identify new targeted therapies and to uncover basic biology relating to SWI/SNF subunit mutation in cancers, rhabdoid tumor cell lines have been the focus of several mechanistic investigations [11,12,13,14]. One major theme that has emerged implicates selective enhancer dysregulation as a fundamental step in driving oncogenic gene expression patterns [1]. For example, in both *SMARCB1*-null rhabdoid tumor cells and *ARID1A*-null colorectal cancer cells, loss of their respective SWI/SNF subunits is associated with a specific collapse of enhancers linked to differentiation and development, while those enhancers driving stemness and renewal remain open and active [11,15,16]. The resulting imbalance in enhancer function thus favors transformation, providing an explanation for how the loss of a single SWI/SNF subunit can, in the absence of other recurring mutations [17], sculpt the transcriptome towards oncogenesis.

Implicit in the above discussion is the notion that in SWI/SNF mutant cancer cells, the remaining SWI/SNF subunits actively maintain open chromatin status at tumor-critical enhancers. Recently, in our study of a kidney-derived *SMARCB1*-null rhabdoid cancer cell line, we reported that BRG1, a synthetic lethal target in rhabdoid cancer cells, actively remodels chromatin at select enhancer regions, and that this remodeling profoundly influences gene expression [18]. We also found that open chromatin sites regulated by BRG1 (here called “BRG1-dependent accessible sites”) show enrichment of DNA sequence motifs bound by AP-1, a signal-responsive transcription factor composed of dimers between the JUN and FOS family of proteins [19]. These studies implicated BRG1 as a key regulatory protein in driving and maintaining rhabdoid gene expression patterns, and AP-1 as a critical factor in this process. Currently, however, the contribution of BRG1-dependent chromatin remodeling to the regulation of the rhabdoid genome and whether additional factors are involved is not clear. Furthermore, whether this is a general phenomenon remains unknown and is particularly important to interrogate experimentally due to the disparate origins and chromatin landscapes of rhabdoid tumor cells [11].

Here, we use a battery of genomic assays in multiple rhabdoid tumor cell lines to dissect the contribution of BRG1 to the regulation of the rhabdoid genome. We find that acute depletion of BRG1 using a specific BRG1/BRM PROTAC (proteolysis-targeting chimera) causes widespread changes to chromatin accessibility and transcriptional patterns. Despite significant heterogeneity at the locus level for BRG1-dependent accessible sites, these regions all share commonalities that implicate AP-1 transcription factor involvement. The biological characteristics of genes regulated by BRG1-dependent chromatin remodeling in rhabdoid tumor cells are remarkably similar, converging on a set of thematically related genes involved in signal transduction, migration, and angiogenesis. Moreover, almost all super-enhancers detected in these rhabdoid tumor lines depend on BRG1 to maintain their open chromatin status. Collectively, these findings shed light on a recurring molecular mechanism contributing to the dysregulation of enhancer- and super-enhancer-mediated gene expression in rhabdoid tumor cells and suggests that AP-1 transcription factors may be underlying factors involved in this process.

## 2. Materials and Methods

### 2.1. Cell Culture and PROTAC Generation

A204 and G401 cells were acquired from ATCC, TTC-549 cells were a gift from Dr. Bernard Weissman, and JMU-RTK-2 cells were provided by the JCRB Cell Bank. Using a Mycoplasma PCR Detection Kit (MP Biomedicals, Irvine, CA, USA), all cell lines were routinely tested for mycoplasma contamination. In addition, cell line integrity was confirmed by STR profiling (ATCC, Manassas, VA, USA). All cell lines were maintained in the appropriate cell maintenance media, either DMEM for G401 and JMU-RTK-2 (Corning) or RPMI-1640 with l-glutamine (A204 and TTC-549) (Corning, Corning, NY, USA), containing 10% fetal bovine serum (FBS) and 1% Penicillin/Streptomycin. For 24 h cell proliferation experiments, 1.0 × 10^6^ cells were plated with 250 nM ACBI1, 250 nM cis-ACBI1, or an equal volume of DMSO, while for four-day cell proliferation experiments, 5.0 × 10^5^ cells were plated similarly. At the end point, cells were counted using an Invitrogen Countess II automated cell counter (Invitrogen, Carlsbad, CA, USA). ACBI1 and cis-ACBI1 were kindly provided by Boehringer Ingelheimvia its open innovation platform opnMe, available at https://opnme.com (accessed on 28 June 2023). Upon arrival, ACBI1 and cis-ACBI1 (Boehringer Ingelheim, ACBI1 and cis-ACBI1, Ingelheim/Rhein, Germany) were dissolved in dimethyl sulfoxide (DMSO).

### 2.2. Western Blotting and Antibodies

Cells were treated with 250 nM ACBI1, cis-ACBI1, or matched DMSO control for 24 h and then harvested for protein lysate preparation. To make lysates, cold lysis buffer containing 5 mM EDTA, 150 mM NaCl, 150 mM Tris, pH 8.0, 1% Triton X-100, 0.001M PMSF, and 1X Roche protease inhibitor cocktail was used to collect the cells after treatment. The cells were sonicated for 15 s at 25% power and centrifuged to remove cellular debris. To determine protein concentration, the BioRad Bradford assay was used with bovine serum albumin as a standard protein. Then, 15–30 μg of protein per sample was separated by SDS-PAGE and then transferred to a PVDF membrane (PerkinElmer, Shelton, CT, USA). Membranes were placed in 5% milk made in TBS-T (150 mM NaCl, 50 mM Tris, 0.1% Tween-20) to block unspecific interactions. The antibodies used for immunoblotting were BRG1 (Cell Signaling, 49360, Danvers, MA, USA), GAPDH-HRP (Cell Signaling, 8884), JUN (Cell Signaling, 9165), JUNB (Cell Signaling, 3753), and JUND (Cell Signaling, 5000). The Clarity ECL substrate (BioRad, Hercules, CA, USA) was used to visualize bands on a Bio-Rad Chemidoc MP instrument.

### 2.3. CUT&RUN

Protocol was performed using the CUT&RUN Assay Kit (Cell Signaling, 72917S and 91931S). A total of 500,000 cells per reaction were collected in 1X Wash Buffer. Cells were then bound to concanavalin A beads and samples were left to rotate overnight at 4 °C in the appropriate antibody. The antibodies used were the negative control IgG and H3K4me3 antibodies included in the CUT&RUN kit, along with H3K4me1 (Cell Signaling, 5326) and H3K27ac (Cell Signaling, 8173). The following day, the samples were resuspended in pAG-MNase pre-mix and left to rotate for 1 h at 4 °C. Calcium chloride was added to activate the pAG-MNase enzyme followed by incubation at 4 °C for 30 min before a 1X Stop Solution was used to end the reaction. The samples were incubated for 10 min at 37 °C and then spun down to collect supernatant. Purification of the DNA was completed by using the Cell Signaling DNA Purification Kit (14209S). An AriaMx Real-Time PCR Machine (Agilent, Santa Clara, CA, USA) was used to quantify the DNA by quantitative PCR using the RPL30 primers included in the kit prior to library generation. Libraries were generated using the NEBNext Ultra II DNA Library Prep Kit for Illumina (Ipswich, Massachusetts, USA, E7645S) and NEBNext Multiple Oligos for Illumina (E6440S0). Libraries were sent to and sequenced at VANTAGE at Vanderbilt University Medical Center on an Illumina NovaSeq6000 with 150 paired-end reads. A minimum of two biological replicates were performed for all CUT&RUN analyses. 

### 2.4. RNA-seq and ATAC-seq

In total, 3.0 × 10^6^ of indicated cells were plated in maintenance media with 250 nM ACBI1, cis-ACBI1, or matched DMSO. Cells were collected in Trizol (Invitrogen, Carlsbad, CA, USA) after 24 h. Using the Direct-zol RNA mini-prep kit (Zymo Research, Irvine, CA, USA), RNA was extracted, and 2 μg was submitted for RNA-Seq to Vanderbilt Technologies for Advanced Genomics (VANTAGE, Nashville, TN, USA) core at Vanderbilt University Medical Center for library generation following ribosomal RNA depletion. An Illumina NovaSeq6000 instrument was used to obtain 150 bp paired-end reads. ATAC-seq was performed as described previously [18]. Briefly, 100,000 cells were harvested and lysed in the ATAC Lysis buffer following 24 h treatment with ACBI1 or matched DMSO control. The following steps including the transposase reaction, generation of the library, and purification of the library were performed according to the manufacturer’s instructions (Active Motif ATAC-Seq kit 53150, Carlsbad, CA, USA). Libraries were submitted to VANTAGE at Vanderbilt University Medical Center and sequenced on an Illumina NovaSeq6000 with 150 paired-end reads. Three biological replicates were performed for ATAC-seq and RNA-seq.

### 2.5. CUT&RUN Analysis

CUT&RUN Tools [20] was used for CUT&RUN data analysis, including adapter trimming, alignment, duplication marking, and peak calling. DiffBind was then applied to obtain consensus peaks for H3K4me3 and H3K27ac [21]. Enhancers were labeled as the sites with H3K27ac and H3K4me1 that did not have H3K4me3 and were more than 1kb away from TSS. Super-enhancers were labeled following the strategy used by Whyte et al. [22]: enhancers within a 12.5 kb were ‘stitched’ together into larger regions. The H3K27ac signal of each of these regions was then determined by the total normalized number reads minus the number of normalized reads in the input. These regions were then sorted by their H3K27ac score, normalized to the highest score and the number of putative enhancer regions. Super-enhancers were identified as regions past the point where the slope was greater than 1. The binding sites of pJUN, JUND, and JUNB were from GSE196960 [23]. To compare the binding intensity between ATAC-seq ACBI1 down sites and other ATAC-seq sites, raw sequencing data for pJUN, JUND, and JUNB were downloaded from GEO and then aligned to the hg19 genome using Bowtie2 [24] after adapter removal.

### 2.6. ATAC-seq Analysis

Adapter trimming was performed using Cutadapt [25] (cutadapt -j -n 3 -q 20 -a CTGTCTCTTATACACATCT -A CTGTCTCTTATACACATC --trim-n), and ATAC-seq reads aligned to the hg19 human genome using Bowtie2 (bowtie2 -p 8 -X 2000 --no-mixed --no-discordant) [24,26]. Peaks were labeled with MACS2 using a q-value of 0.05 [27]. Peaks with a minimum of two replicates per condition were included, and all peaks identified across conditions were then combined into a final peak set. ATAC-seq read counts were calculated for the final peak set. Differentially enriched peaks were identified using DiffBind with an FDR < 0.05 [21]. The HOMER program ‘annotatePeaks.pl’ was used for peak annotation, and ‘findMotifsGenome.pl’ was used for identification of enriched motifs.

### 2.7. RNA-seq Analysis

Adapter trimming was performed using Cutadapt [25], (cutadapt -j 2 -q 20 -a AGATCGGAAGAGCACACGTC -A AGATCGGAAGAGCGTCGTGT -m 30 -n 3 --trim-n) and reads aligned to the hg19 human genome using STAR [28] and quantified by featureCounts [29]. Differential analysis was performed using DESeq2 [30] and adjusted *p*-value (FDR) was determined by the Benjamini–Hochberg procedure. Significantly changed genes were identified with an FDR < 0.05. The hypergeometric test was performed on the gene groups in the indicated Venn diagrams using the R functions phyper and dhyper. 

## 3. Results

### 3.1. Acute Depletion of BRG1 Using the ACBI1 Degrader Causes Widespread Gene Expression Changes in Rhabdoid Cell Lines

**Figure 1:**
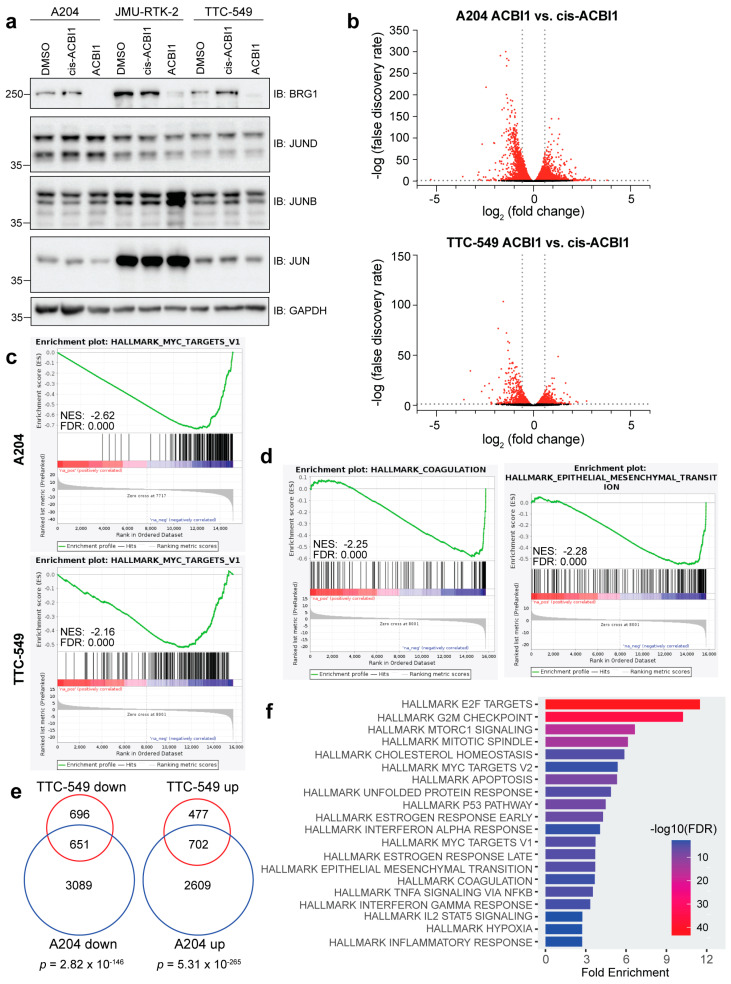
BRG1 regulates the expression of genes associated with important cellular processes. (**a**) Western blot showing the effect of 250 nM ACBI1 treatment when compared to 250 nM cis-ACBI1 or matched DMSO control. Indicated cell lines were treated for 24 h and protein lysates probed for BRG1, JUN, JUNB, and JUND. GAPDH is present as a loading control for samples. (**b**) Volcano plot showing magnitude and significance of all genes detected in RNA-seq analysis between ACBI1 vs. cis-ACBI1 for A204 or TTC-549 cells. Red indicates significantly changed genes in either direction (FDR < 0.05). Grey lines mark an FDR of 0.05 and fold change of 1.5. (**c**) Gene set enrichment analysis was performed using ACBI1 vs. cis-ACBI1 gene expression changes and the MSigDB hallmark data sets for indicated cell lines. Genes were negatively enriched within the “MYC targets V1” gene set with normalized enrichment score (NES) and FDR shown. (**d**) TTC-549 GSEA results show differentially changed genes were negatively enriched within the “Coagulation” and “Epithelial-mesenchymal transition” gene sets. (**e**) Venn diagram comparing unique genes decreased or increased in ACBI1 vs. cis-ACBI1 RNA-seq analysis between cell lines (FDR < 0.05). Significance of overlap between gene groups is displayed and was determined by performing a hypergeometric test. (**f**) GO analysis of 651 genes that were commonly decreased in expression from (**e**). n = three independent RNA-seq experiments performed for each cell line. The uncropped blots are shown in Figure S1.

In our previous work, we identified thousands of BRG1-dependent genes in kidney-derived G401 cells following 24 h depletion of BRG1 with a chemical degrader called ACBI1 [18]. Because rhabdoid tumors can originate in various tissues, including kidney, liver, brain, and soft tissue [31], we performed RNA-seq following BRG1 depletion for 24 h in two additional cell lines—A204 (soft-tissue-derived) and TTC-549 (liver-derived)—to understand the totality of genes regulated by BRG1. We selected these two cell lines to both broaden our understanding of BRG1 gene regulation beyond kidney-derived rhabdoid cells and have representative data from cells derived from three of the four known tissue origins for any comparisons. To control for any off-target effects, we also profiled cis-ACBI1, a distomer of ACBI1 [32]. Treatment with 250 nM ACBI1 compared to 250 nM cis-ACBI1 or dimethyl sulfoxide (DMSO) for 24 h led to the depletion of BRG1 while not overtly affecting the levels of JUN family members, except for an increase in JUNB protein levels noted in a separate kidney-derived JMU-RTK-2 cell line we tested (Figure 1a and Figure S1). Important for capturing early gene expression changes, at 24 h, no obvious impact to cellular proliferation was observed in the A204 or TTC-549 cells (Figure S2a,b). RNA-seq analysis at this same timepoint for cis-ACBI1-treated samples compared to DMSO-treated samples shows that only two genes in A204 and none in the TTC-549 cells are significantly changed in response to the distomer (false discovery rate, FDR < 0.05) (Figure S2c). In contrast, the analysis of ACBI1-treated samples compared to cis-ACBI1-treated samples revealed thousands of gene expression changes (FDR < 0.05) in either direction (Figure 1b, Table S1). The profound differences between ACBI1 and cis-ACBI1 indicate that the effects of using ACBI1 are specific to induced degradation, in line with the reported high specificity of ACBI1 [32]. For each cell line, gene set enrichment analysis (GSEA) performed using the MSigDB Hallmark data sets showed that suppressed genes cluster within more biological categories than genes that increased in expression (Table S2). Gene sets related to “MYC targets” were among the top categories based on FDR score (Table S2, Figure 1c), supporting the idea that various SWI/SNF subunits can modulate MYC activity and function [33,34]. In addition, suppressed genes are also enriched within functions related to cell cycle processes including “Mitotic Spindle”, “E2F targets”, and “G2/M checkpoint” (Table S2, Figure S2d,e). In TTC-549 cells, suppressed genes showed enrichment in unique categories such as “Coagulation” and “Epithelial Mesenchymal Transition” (Figure 1d), which are present in the A204 GSEA results, albeit with a lower normalized enrichment score (NES) and higher FDR value (Table S2). Direct comparison of differentially expressed genes between cell lines reveals that ~50% of genes changed in TTC-549 cells are identical to those changed in A204, an overlap that was highly significant based on hypergeometric test results (Figure 1e). A comparison of commonly repressed genes against Hallmark MSigDB data sets using ShinyGO analysis [35] confirms that common genes are enriched within similar biological categories (Figure 1f). Finally, we asked how BRG1 depletion impacts the expression of known or likely transcription factors by comparing a list of high-confidence transcription factors [36] to the list of differentially expressed genes in each cell line. We find that approximately 50% of the transcription factors that change in expression in TTC-549 cells also change in A204 cells (Figure S2f). Transcription factors related to cell cycle control and DNA damage and repair (such as *E2F2*, *E2F7*, *E2F8*, *CDC5L*, *CENPA*, *HMGA2*, and *LIN54*) are commonly decreased in expression, while those related to developmental processes such as *MEIS3*, *HAND2*, *HES2*, *HES4*, *HOXA1*, *HOXD9*, *HOXD8*, and *DOXD9* are commonly increased (Figure S2g). Taken together, these data indicate that while rhabdoid cell lines have rather disparate chromatin landscapes [11], BRG1-dependent genes cluster within similar functional categories and include many key transcription factors.

### 3.2. BRG1-Dependent Accessible Sites Detected across Diverse Rhabdoid Cell Lines Implicate Involvement of the AP-1 Transcription Factor

**Figure 2:**
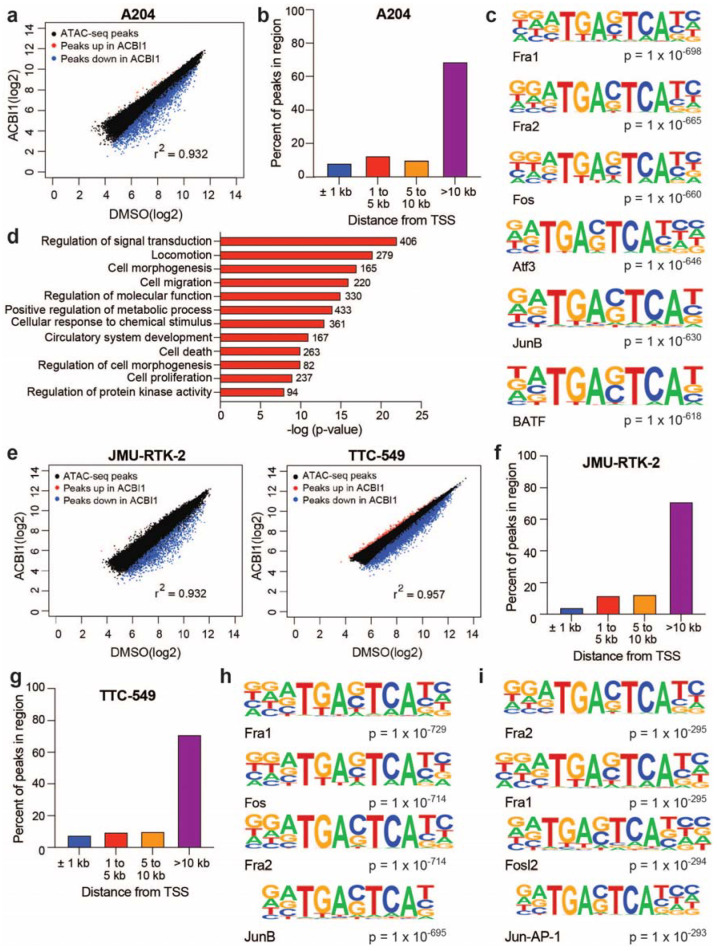
BRG1-dependent accessible sites are enriched in AP-1 subunit motifs across diverse rhabdoid cell lines. (**a**) Scatterplot of log_2_-fold values for ATAC peaks across DMSO vs. ACBI1 samples in the A204 rhabdoid cell line. Blue color represents ATAC peaks with a decrease in peak intensity upon ACBI1 treatment, while the red color represents ATAC peaks that were increased in peak intensity. (**b**) All significantly changed ATAC peaks (FDR < 0.05) in A204 cells were annotated and distance of each peak to the nearest TSS was determined. Bars show the percent of total peaks in each region. (**c**) Known motif analysis results for all decreased ATAC peaks in A204 cells. Top six motifs are shown. (**d**) All significantly changed ATAC peaks detected in A204 cells (FDR < 0.05) were annotated to their nearest gene and subsequent GO-term analysis performed on unique genes. The number of genes in each category are noted next to the bar and significance of enrichment within each category is on the *x*-axis. (**e**) Scatterplot of log_2_-fold values for ATAC peaks in the indicated cell lines. Blue color represents ATAC peaks with a decrease in peak intensity upon ACBI1 treatment, while the red color represents ATAC peaks that were increased in peak intensity. All significantly changed ATAC peaks (FDR < 0.05) in JMU-RTK2 (**f**) or TTC-549 cells (**g**) were annotated and the distance of each peak to the nearest TSS was determined. Bars show the percent of total peaks in each region. (**h**) Known motif analysis results for decreased ATAC peaks in JMU-RTK-2 cells. Top four motifs are shown. (**i**) Similar analysis as in (**h**) was performed for TTC-549 cells (fold change < −1.5). n = three independent ATAC-seq experiments performed for each cell line.

To understand how active chromatin remodeling influences DNA accessibility across rhabdoid genomes, we performed Assay for Transposase-Accessible Chromatin coupled to next-generation sequencing (ATAC-seq) in the A204, TTC-549, and JMU-RTK-2 (kidney-derived) cell lines following degradation of BRG1. In A204 cells, we detected ~50,000 total open sites of chromatin (“ATAC-peaks”), ~4100 of which decreased within 24 h of BRG1 degradation (FDR < 0.05, fold change < −1.5) (Table S3, Figure 2a). Thus, as we saw in the kidney-derived G401 cell line [18], BRG1 maintains DNA accessibility in A204 cells. Interestingly, depletion of BRG1 shows a strong bias for impacting TSS-distal sites (Figure 2b), even though many ATAC peaks are detected near promoters (Figure S3a). Known motif analysis of BRG1-dependent accessible sites shows these sites are heavily enriched in motif sequences of AP-1 subunits (Figure 2c, Table S4). Annotation of BRG1-dependent sites to their nearest gene and subsequent gene ontology (GO) analysis (https://david.ncifcrf.gov/ (accessed on 12 July 2023) shows that BRG1-dependent accessible sites are annotated to genes enriched within functions involving signaling, cell death, cell proliferation, migration, and angiogenesis (Figure 2d).

Consistent with A204 cells (Figure 2a), in the JMU-RTK-2 and TTC-549 cell lines, the predominate response upon BRG1 depletion is a decrease in open chromatin sites with ~1700 and ~5500 ATAC peaks impacted in JMU-RTK-2 and TTC-549 (Figure 2e), respectively (FDR < 0.05, fold change < −1.5) (Table S3). In addition, BRG1-dependent accessible sites were localized at transcription start site (TSS)-distal regions of the genome (Figure 2f,g), even though TSS-proximal peaks are represented among all ATAC-seq sites detected (Figure S3b,c). Strikingly, known motif analysis of BRG1-dependent accessible sites once again revealed enrichments of AP-1 subunit motifs regardless of cell line (Figure 2h,i, Table S4) and GO analysis of annotated genes points to highly similar functional categories (Figure S3d,e), revealing that BRG1-dependent accessible sites in rhabdoid genomes share a variety of key features. 

Next, we compared BRG1-dependent accessible sites identified in our ATAC-seq analysis to changes in gene expression detected in our RNA-seq analysis to determine how many genes at this timepoint may be regulated by BRG1-mediated chromatin remodeling. In TTC-549 cells, ~36% of repressed genes significantly overlap with genes annotated to BRG1-dependent accessible sites (Figure S4a), while only ~7% of induced genes show any overlap and are not significant using a hypergeometric test. GO-term analysis of the 487 genes that annotate to BRG1-dependent accessible sites and have a concordant change in gene expression highlight gene categories related to signal transduction and migration as potential biological processes regulated by BRG1-dependent chromatin remodeling (Figure S4b). In A204 cells, ~17% of repressed genes overlap significantly with BRG1-dependent accessible sites, which is about three times more than that of induced genes (Figure S4c). Like TTC-549 cells, GO-term analysis of the 642 genes that annotate to BRG1-dependent accessible sites and have a concordant change in gene expression shows that these genes are enriched within biological functions related to signal transduction, movement and migration, and development (Figure S4d). Taken together, these data provide compelling evidence that BRG1-dependent chromatin remodeling mechanistically impacts expression of specific types of genes that are thematically related across rhabdoid cell lines.

### 3.3. BRG1-Dependent Accessible Sites Show JUN Family Member Binding

**Figure 3:**
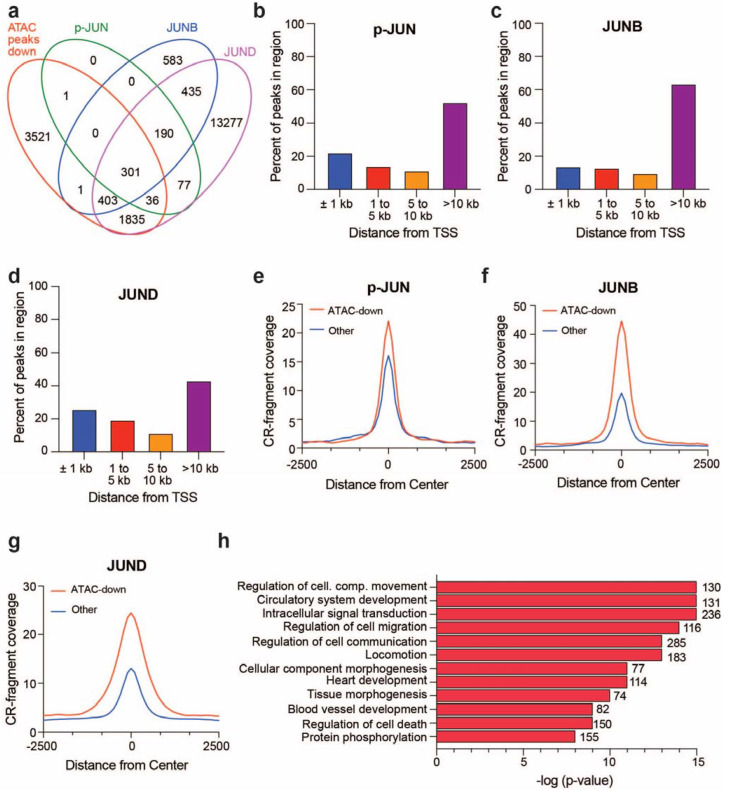
JUN family member binding correlates with BRG1-dependent changes in chromatin accessibility. (**a**) Venn diagram comparing ATAC peaks that significantly decrease (FDR < 0.05) in G401 cells upon depletion of BRG1 for 24 h [18] compared to phosphorylated JUN (p-JUN-Ser73), JUNB, and JUND chromatin binding sites detected separately through CUT&RUN assays [23]. Approximately 37% of JUNB, 15% of JUND, and 56% of p-JUN binding sites overlap with an ATAC-seq site that shows a BRG1-dependent change in accessibility. (**b**) p-JUN binding sites detected were annotated to determine the distance of binding sites to their nearest TSS. Percentages of p-JUN localized within each bin are shown in bar graph. Similar analysis was performed for JUNB (**c**) and JUND (**d**). (**e**) Average normalized p-JUN CUT&RUN (“CR”) signal at BRG1-dependent accessible sites (FDR < 0.05) in G401 cells compared to all other binding sites detected. Similar analysis was performed for JUNB (**f**) and JUND (**g**). (**h**) Gene ontology analysis was performed on genes that were both associated with JUND chromatin binding and were BRG1-dependent accessible sites. The number of genes in each category are noted next to the bar and significance of enrichment within each category is on the x-axis.

An interesting observation in the analysis of BRG1-dependent accessible sites across G401 [18], A204, JMU-RTK-2, and TTC-549 cells is the enrichment of AP-1 transcription factor motifs, suggesting AP-1 chromatin binding at BRG1-regulated genomic sites. Given the well-documented connection between AP-1 and SWI/SNF [23,37]—and the described role of AP-1 in the regulation of genes involved in signal transduction and migration—we next assessed AP-1 residency at BRG1-dependent accessible sites. For this, we focused on G401 cells, a cell line in which we have ATAC-seq data and publicly available JUN family member chromatin binding data collected using Cleavage Under Targets & Release Using Nuclease (CUT&RUN) assays [23]. We find that of the 6098 BRG1-dependent accessible sites detected in G401 cells, 2577 overlap with binding sites for JUN phosphorylated at Ser-73 (p-JUN), JUNB, or JUND (Figure 3a). In addition, analysis of the location of binding sites for all JUN family members shows the highest binding at TSS-distal regions (Figure 3b–d), consistent with BRG1-dependent accessible sites being predominantly TSS-distal [18]. Interestingly, the analysis of normalized CUT&RUN signals for each JUN family member at BRG1-dependent accessible sites versus all other sites shows larger p-JUN, JUNB, and JUND signal at sites that are BRG1-regulated (Figure 3e–g). This is especially prominent for JUNB and JUND where the amount detected on chromatin at BRG1-dependent accessible sites is over double that present at all other sites (Figure 3f,g). Across rhabdoid cell lines, BRG1-dependent accessible sites are associated with genes enriched in various cancer-relevant biological functions, such as signaling, migration, and angiogenesis (Figure 2). To determine if these gene categories are also associated with chromatin-bound JUN family members, we focused on JUND because most p-JUN and JUNB binding sites are also cobound by JUND and almost all BRG1-dependent accessible sites include colocalization with at least JUND (Figure 3a). Annotation and subsequent GO-term analysis of JUND binding sites that are also BRG1-dependent accessible sites again highlight these specific sets of genes (Figure 3h). Overall, these data provide support for the notion that BRG1 regulates accessibility at TSS-distal regions bound by AP-1 subunits in rhabdoid tumor cells.

### 3.4. BRG1-Dependent Accessible Sites Are Context-Dependent

**Figure 4:**
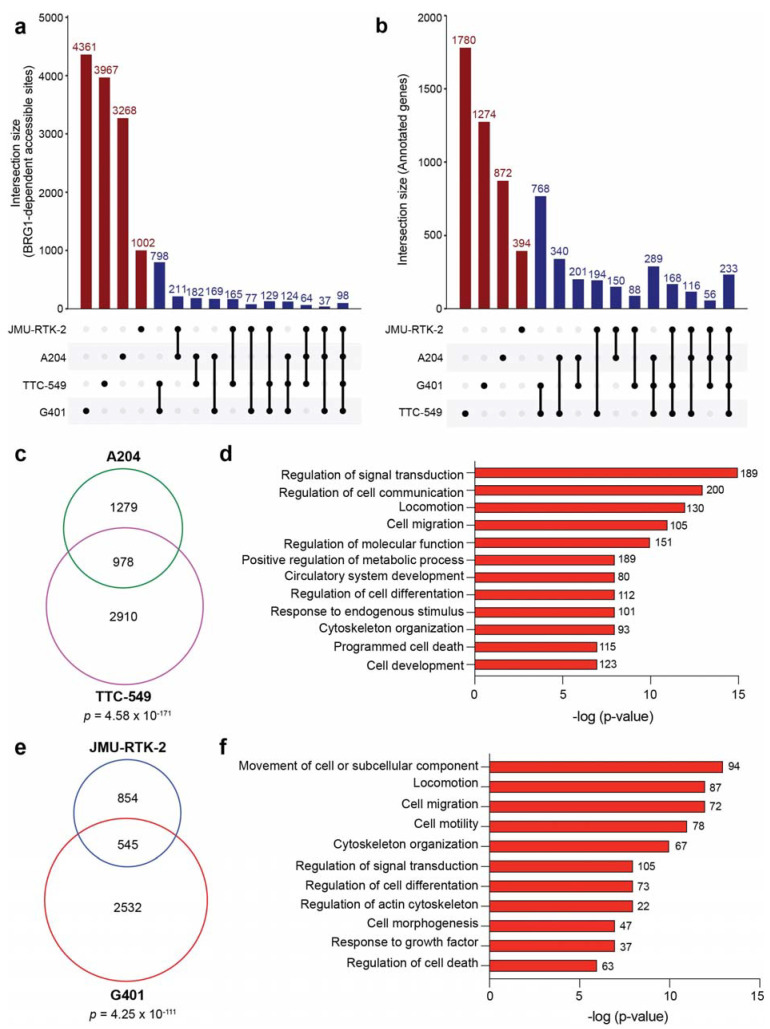
BRG1 maintains open chromatin at context-dependent sites. (**a**) UpSet plot [41,42] comparing the overlap of BRG1-dependent accessible sites (FDR < 0.05, fold change < −1.5) detected among all rhabdoid cell lines indicated. Red bars show the number of sites in individual cell lines with no overlap and blue bars show the various overlaps of detected sites between cell lines, as denoted by a connection line drawn below. Overlap was determined if ATAC-seq peaks overlapped by at least one base pair. (**b**) UpSet plot comparing genes identified in each cell line through annotating BRG1-dependent accessible sites (FDR < 0.05) to their nearest gene. Bars are indicated as described in (**a**). (**c**) Venn diagram comparing unique genes annotated from (**b**) for A204 and TTC-549 cells. The significance of overlap between the gene sets is displayed and was determined using a hypergeometric test. (**d**) GO-term analysis performed on 978 genes from overlap in (**c**). The number of genes in each category are noted next to the bar and the significance of enrichment within each category is on the x-axis. (**e**) Venn diagram comparing unique genes from (**b**) for JMU-RTK-2 and G401 cells. The significance of overlap is shown. (**f**) GO-term analysis performed on 545 genes from overlap in (**e**). The number of genes in each category are noted next to the bar and the significance of enrichment within each category is on the x-axis.

For many characteristics assessed, BRG1-dependent accessible sites share several common features regardless of cell line. Therefore, we sought to determine how conserved BRG1-regulated sites are across rhabdoid cells. By analyzing the overlap between BRG1-dependent accessible sites (FDR < 0.05, fold change < −1.5) detected in G401, A204, JMU-RTK-2, and TTC-549 cells, we observe that less than 100 sites are common to all four cell lines at the loci level (Figure 4a). Even in the case of G401 and JMU-RTK-2, which are both categorized as kidney-derived, less than 20% of BRG1-dependent accessible sites in JMU-RTK-2 are shared in G401 cells. Because TSS-distal regulatory sites such as enhancers can be tumor-specific but still converge on common genes [38,39], we also determined if genes annotated to BRG1-dependent accessible sites (FDR < 0.05) show any commonalities. In performing this gene comparison, it is still clear that across all four cell lines there is little conservation between all rhabdoid tumor cell types (233 genes), However, there does exist a larger overlap when looking between specific cells (Figure 4b). For example, between soft-tissue-derived A204 and liver-derived TTC-549 cells, ~43% of genes are shared (Figure 4c). In addition, ~39% of annotated genes in kidney-derived JMU-RTK-2 cells are also annotated in G401 cells (Figure 4e). This finding suggests that while BRG1-dependent accessible sites are predominantly cell-type-specific, the types of genes they converge on are remarkably similar. To further explore this idea, we performed GO-term analysis on the 978 genes shared between A204 and TTC-549 cells and the 545 genes shared between JMU-RTK-2 and G401 cells (Figure 4c,e), some of which are also shared across all cell lines (Figure 4b). As shown in Figure 4d,f, many of the conserved genes are enriched within common biological functions related to cell migration, motility, signal transduction, and cell differentiation. The only distinct gene ontology terms that separate the two analyses are linked to angiogenesis and circulatory system development, which were identified solely in the A204 and TTC-549 comparison. As a second approach, we also performed over-representation analysis on the 978 and 545 common genes to identify the top 10 enriched KEGG and Reactome pathways. Similar to the gene ontology analysis, the pathways identified include those related to signaling and cytoskeletal function (Table S5). We conclude based on these data that despite significant heterogeneity at the locus level, the biological characteristics of genes regulated by BRG1-dependent chromatin remodeling in rhabdoid tumor cells are strikingly similar and converge on a set of thematically related genes and pathways. Furthermore, given that the common gene categories are known biological functions attributed to the AP-1 transcription factor [40] (Figure 3h), these data provide additional evidence that AP-1 may be involved in gene regulation by SWI/SNF in rhabdoid tumor cells. 

### 3.5. Accessible Sites Correlate with Enhancer Regions

**Figure 5:**
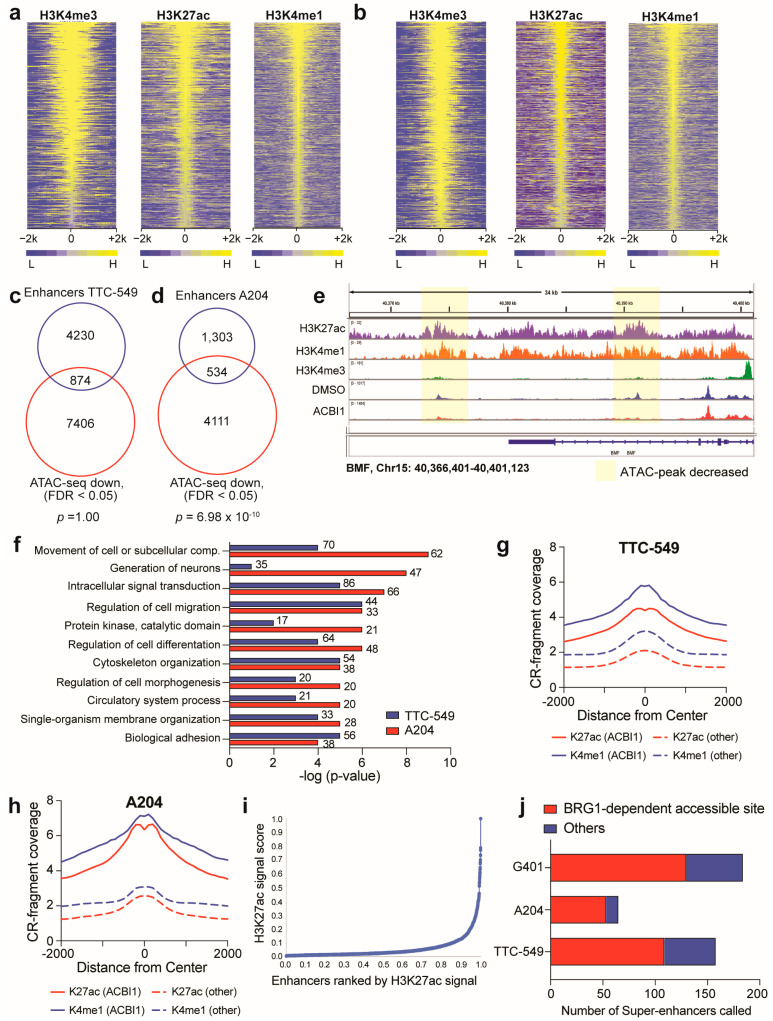
Accessible sites correlate with enhancers. (**a**) Heatmap showing normalized CUT&RUN peak intensity for H3K4me3, H3K4me1, and H3K27ac detected genome-wide in TTC-549 cells. Yellow color depicts signal in 100 bp bins within 2 kb from center of peaks. (**b**) Heatmap showing normalized CUT&RUN peak intensity for H3K4me3, H3K4me1, and H3K27ac detected genome-wide in A204 cells. Yellow color depicts signal in 100 bp bins within 2 kb from center of peaks. (**c**) Venn diagram showing the overlap between active enhancers identified in TTC-549 cells and all ATAC-seq sites that decreased (FDR < 0.05). Significance of overlap was tested by performing a hypergeometric test. (**d**) Venn diagram showing the overlap between active enhancers identified in A204 cells and all ATAC-seq sites that decreased (FDR < 0.05). Significance displayed is the value obtained by performing a hypergeometric test. (**e**) Example genome browser tracks in IGV [43] showing ATAC-seq sites that decrease upon ACBI1 treatment and overlap with H3K27ac/H3K4me1-marked enhancers. View shown is 34 kb stretch of the region at the BCL2 modifying factor (BMF) gene from TTC-549 cell data. (**f**) The 874 peaks in (**c**) and 534 peaks in (**d**) were annotated to their nearest gene and GO analysis was performed on unique genes to look for common gene categories. The number of genes in each category are noted next to the bar and the significance of enrichment within each category is on the x-axis. The abbreviation “comp.” means “compartment”. (**g**) Average normalized H3K27ac and H3K4me1 CUT&RUN (“CR”) signal at ATAC-seq sites that had a decrease in accessibility upon BRG1 depletion (“ACBI1”, FDR < 0.05) compared to all ATAC-seq sites (“other”); data shown for TTC-549 cells. Similar analysis was performed in (**h**) for A204 cells. (**i**) Graph showing an example of how super-enhancers were identified based on ranking of H3K27ac signal across all enhancers; data shown for TTC-549 cells. All the regions past the point where the slope was greater than 1 are considered “super-enhancers”. (**j**) Super-enhancers were identified and compared to ATAC-seq data to determine any overlap with BRG1-dependent accessible sites. Data plotted as a stacked bar graph for visualization of differences. n = minimum two independent CUT&RUN experiments performed for each cell line.

The propensity of BRG1-dependent accessible sites to be localized at TSS-distal regions (Figure 2) and be cell-type-specific (Figure 4) suggest that at least some of these sites may be enhancers. To determine if this is true, we performed CUT&RUN for three histone marks: histone 3 lysine 4 trimethylation (H3K4me3), histone 3 lysine 4 monomethylation (H3K4me1), and histone 3 lysine 27 acetylation (H3K27ac) in A204 and TTC-549 cells (Figure 5a,b). We identified enhancers from promoters by separating out binding sites that were positive for H3K4me3 and H3K27ac (promoters) from those that relatively lacked H3K4me3 but contained H3K4me1 and H3K27ac and were more than 1 kb from a promoter (enhancers). The overlap of the identified enhancer regions with BRG1-dependent accessible sites (FDR < 0.05) shows that approximately 20–30% of identified enhancers show a decrease in chromatin accessibility when BRG1 is depleted (Figure 5c–e), a proportion similar to what we observed in G401 cells previously [18]. In both cell lines, overlapped regions are annotated to genes associated with various common biological functions already identified in this study, such as genes involved in migration and signaling (Figure 5f) and BRG1-dependent accessible sites occurring at enhancers are marked by higher H3K27ac and H3K4me1 signal (Figure 5g,h), suggesting the involvement of enhancers in the regulation of these genes. Because of differences in H3K27ac/H3K4me1 signals at enhancers that respond to BRG1 loss, we wanted to further dissect these data by separating out super-enhancers, which can be distinguished from typical enhancers based on size, transcriptional potential, and density of chromatin resident proteins [22,38]. Importantly, in *SMARCB1*-null rhabdoid cancer cells, super-enhancers are both bound by remaining SWI/SNF subunits and are essential for rhabdoid tumor cell survival [11]. Therefore, it is possible that BRG1 regulates super-enhancer function, providing a mechanism by which retained SWI/SNF subunits can modulate super-enhancer function to maintain the rhabdoid cancer state. To determine any relationship between BRG1-dependent accessible sites and super-enhancers, we ranked all enhancers based on intensity of H3K27ac and labeled super-enhancers as those with the highest H3K27ac (Figure 5i). Using this method, 158, 65, and 184 super-enhancers were identified in TTC-549, A204, and G401 cells, respectively, although comparison of any overlap between these super-enhancers reveals the cell lines only share three super-enhancer regions among them. Of all super-enhancers detected, approximately 70–80% show a BRG1-dependent change in accessibility upon ACBI1 treatment (Figure 5j), pointing to a regulatory role for BRG1 at super-enhancers. In sum, BRG1-dependent chromatin remodeling regulates DNA accessibility at distinct genomic sites that correlate with histone-marked enhancers, with a considerable impact present at super-enhancers. Collectively, these findings suggest that BRG1 plays a role in maintaining super-enhancer function through active chromatin remodeling.

## 4. Discussion

Dysregulation of enhancer-mediated gene expression in cancers defined by SWI/SNF subunit mutation is a two-step process involving the collapse of enhancers controlling the expression of genes needed for differentiation and development while allowing other enhancers to stimulate an overtly oncogenic gene expression program [1]. Contributing to enhancer dysregulation is the influence that remaining SWI/SNF subunits have in changing SWI/SNF from functioning in tumor suppression to instead functioning as a driver of pro-tumorigenic gene expression programs. In *SMARCB1*-null rhabdoid tumors, the presence of BRG1 and the non-canonical SWI/SNF subunit, BRD9, are synthetic lethal targets in rhabdoid cancers [14,44], although the mechanisms by which they maintain a rogue gene expression program are not entirely clear. 

In this study, we examine the extent to which BRG1 regulates gene expression and DNA accessibility across rhabdoid cancer cell genomes. Using transcriptome analysis, we first find that BRG1-regulated genes are highly similar regardless of rhabdoid cancer cell context (Figure 1). To understand how BRG1 regulates DNA accessibility across the genomes of rhabdoid cell lines, we performed chromatin accessibility assays to identify BRG1-dependent accessible sites. Strikingly, accessible sites regulated by BRG1 share a variety of common characteristics, including TSS-distal localization, enrichment of AP-1 transcription factor motifs, and convergence on thematically related genes involved in signal transduction, migration, angiogenesis, and development (Figure 2). The identification of AP-1 motifs within BRG1-accessible sites suggests the presence of chromatin-bound AP-1, which we confirmed by analyzing JUN family member chromatin binding (Figure 3), indicating that AP-1 may be involved in BRG1-dependent regulation of these genomic regions. AP-1 has the potential to be pro- or anti-tumorigenic depending on context [19] and at least in *ARID1A*-null colorectal cancer cells, changes in genome regulation implicate AP-1 in controlling new gene networks associated with the regulation of oncogenic gene expression [15]. At least as it relates to rhabdoid tumors, AP-1 involvement in pro-tumorigenic processes is a novel concept and could be related to the ability of AP-1 to recruit SWI/SNF to enhancers, albeit the enhancers in this case are not being used for the completion of differentiation and development as is the accepted paradigm [23,37]. Therefore, it is possible that in the background of improper SWI/SNF complex function, AP-1, like BRG1, can function to favor the expression of genes that facilitate and maintain cancer cell identity. On the other hand, it is also possible that AP-1 binding and regulation of chromatin accessibility by SWI/SNF complexes is non-oncogenic in nature and the presence of AP-1 subunits on chromatin marks the regions of the genome that were once part of a functional AP-1-SWI/SNF gene network. As it was recently shown that the AP-1 subunit JUN dramatically increases both in protein levels and chromatin binding upon reintroduction of *SMARCB1* [23], it may be that changes in JUN expression are an underlying mechanism by which the AP-1-SWI-SNF gene network becomes impaired. 

The localization of BRG1-dependent accessible sites to TSS-distal regions and their cancer cell specificity at the loci level (Figure 2 and Figure 4) prompted us to determine how many BRG1-dependent accessible sites are enhancer regions, which we accomplished through performing chromatin-binding assays for histone proteins to delineate promoters versus enhancers in the genome. The fraction of sites that are marked as enhancers are low in comparison to all BRG1-dependent accessible sites but contain defining characteristics such as increased H3K27ac signal and annotation to genes involved in a variety of biological functions such as development, signaling, and migration (Figure 5). In *SMARCB1*-null rhabdoid cells, the enhancers that stimulate the expression of genes associated with increased cancer function are so-called super-enhancers [11] which are known to be important in regulating cell identity, much like that of typical enhancers, and are particularly prevalent in cancer where they can be acquired at tumor-specific oncogenes during the route to tumor formation [38,39]. Therefore, we specifically looked at these larger enhancer regions and found that the majority of super-enhancers detected across three rhabdoid cancer cell lines show changes in DNA accessibility upon BRG1 depletion (Figure 5), pointing to BRG1-dependent chromatin remodeling being a key factor in maintenance of super-enhancer function. While our current study was focused on multiple cell lines derived from three of the four known origins for rhabdoid tumors (kidney, liver, and soft tissue), a recent comprehensive analysis of childhood pediatric cancers established *SMARCA4* as a dependency in atypical teratoid rhabdoid tumors (ATRTs) [45]. It is certainly possible that the results of this study could be relevant for understanding the contribution of BRG1 to ATRT-specific enhancer regulation and future studies aimed at determining the mechanisms by which ATRTs are dependent on BRG1 expression should be very informative.

## 5. Conclusions

Overall, our study set out to determine the contribution of BRG1-dependent chromatin remodeling to rhabdoid genome regulation and in doing so uncovered important biological results to consider when thinking of how dysregulated enhancer function is achieved in these tumors. First, these data point to a unique interplay between AP-1 and SWI/SNF that has yet to be fully investigated but suggests that the AP-1-SWI/SNF interaction can select for enhancers during tumor evolution. Second, the thematically related genes we continually identified across our genomic assays have ties to known AP-1-regulated genes and therefore AP-1 may be a good candidate to investigate as a new target in rhabdoid cancers, particularly as it relates to migration and angiogenic tumor functions. Lastly, BRG1-dependent chromatin remodeling has a prominent effect on the regulation of DNA accessibility at super-enhancer regions. Strategies to target super-enhancer function—which are actively pursued in the cancer field [46]—may be effective in rhabdoid tumors or other cancers in which SWI/SNF subunit mutation occurs.

## Data Availability

Sequencing data relevant to A204, TTC-549, and JMU-RTK-2 cells were deposited on GEO with the accession number GSE241874. Sequencing metrics are included in Table S6. G401 ATAC-seq data were previously published under GSE198156 [18] and G401 CUT&RUN data for AP-1 subunits were previously published under GSE196960 [23]. Upon request, additional data are available.

## Supplementary Materials

The following supporting information can be downloaded at: https://www.mdpi.com/article/10.3390/cancers16050916/s1, Figure S1: Uncropped Western blots; Figure S2: Transcriptome analysis of diverse rhabdoid cell lines; Figure S3: Additional ATAC-seq analysis of diverse rhabdoid cell lines; Figure S4: Comparison of RNA-seq and ATAC-seq analysis in TTC-549 and A204 cell lines; Table S1: Differentially expressed genes identified in ACBI1-treated samples compared to cis-ACBI1-treated samples in indicated cell lines; Table S2: Gene set enrichment analysis against MSigDB Hallmark datasets (ACBI1 vs. cis-ACBI1); Table S3: Differential analysis and annotation of all ATAC-peaks that showed a significant change in intensity following treatment with ACBI1 for 24 h; Table S4: Known motif analysis results for ATAC-seq peaks that significantly decreased with ACBI1 treatment in indicated cell lines; Table S5: Over-representation analysis of common genes annotated to BRG1-dependent accessible sites; Table S6: Sequencing metrics.
