# Supplementary material for: Super-Enhancer Dysregulation in Rhabdoid Tumor Cells Is Regulated by the SWI/SNF ATPase BRG1
Source: Cancers (Basel). 2024 Feb 24;16(5):916. doi: 10.3390/cancers16050916 (PMC10931202; doi:10.3390/cancers16050916)
Supplement: Supplementary file 1 [file cancers-16-00916-s001.zip › Supplementary Material_Revised.pdf]

Supplementary Materials

Super-enhancer dysregulation in rhabdoid tumor cells is regulated by the SWI/SNF ATPase BRG1

Jones, CA et al.

Included figures:

Figure S1. Uncropped Western blots.

Figure S2. Transcriptome analysis of diverse rhabdoid cell lines.

Figure S3. Additional ATAC-seq analysis of diverse rhabdoid cell lines.

Figure S4. Comparison of RNA-seq and ATAC-seq analysis in TTC-549 and A204 cell lines.

Excel files:

Table S1. Differentially expressed genes identified in ACBI1-treated samples compared to cis-ACBI1-treated samples in indicated cell lines. RNA-seq results for A204 and TTC-549 cells (ACBI1 versus cis-ACBI1).

Table S2. Gene set enrichment analysis against MSigDB Hallmark datasets (ACBI1 vs. cis-ACBI1). Gene set enrichment analysis results for indicated cell lines. RNA-seq data was compared to the MSigDB Hallmark data sets.

Table S3. Differential analysis and annotation of all ATAC-peaks that showed a significant change in intensity following treatment with ACBI1 for 24 hr.

ATAC-peaks that significantly changed in ACBI1-treated samples versus DMSO-treated samples are shown with annotation of peaks performed using Homer.

Table S4. Known motif analysis results for ATAC-seq peaks that significantly decreased with ACBI1 treatment in indicated cell lines. Motif analysis for ATAC-peaks that significantly changed (FDR < 0.05) in ACBI1-treated samples versus DMSO-treated samples. For TTC-549 cells, a FDR < 0.05 and fold change > 1.5 fold was used.

Table S5. Over-representation analysis of common genes annotated to BRG1-dependent accessible sites. Over-representation analysis results showing top ten KEGG and Reactome pathways that were determined for the 978 annotated genes common between A204 and TTC-549 cells (Figure 4c) or the 545 annotated genes common between G401 and JMU-RTK-2 cells (Figure 4e).

Table S6. Sequencing metrics. General sequencing metrics for all fastq files that were used in this study.

Chemiluminescence images used

Merged colormetric image with ladder marked in red

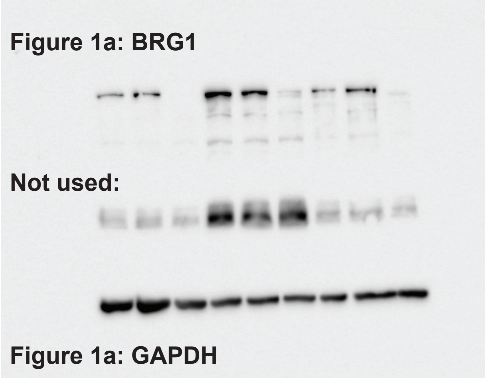

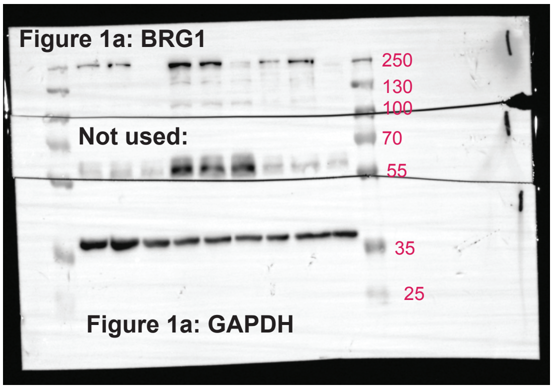

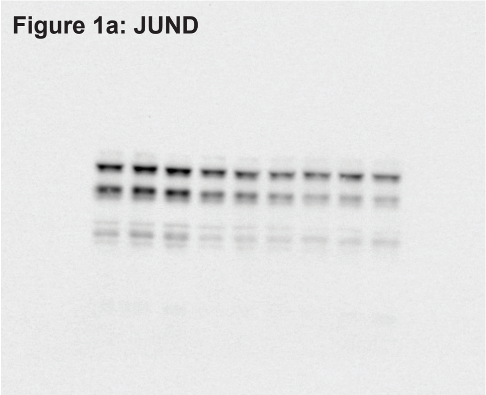

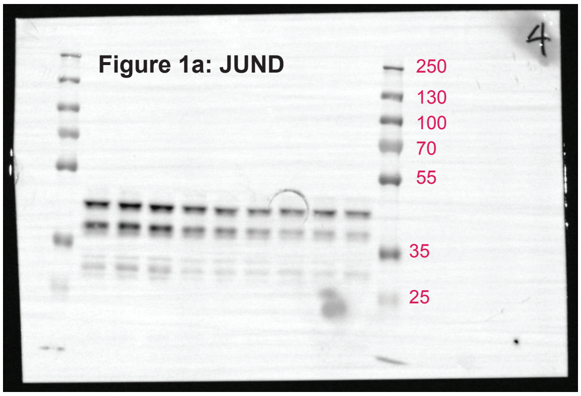

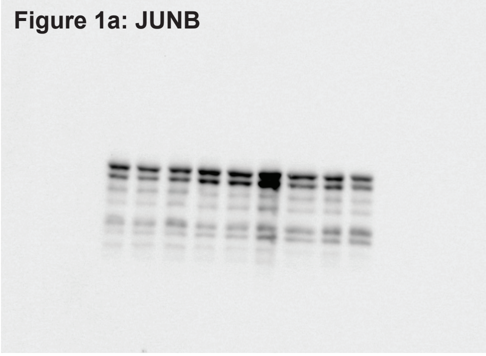

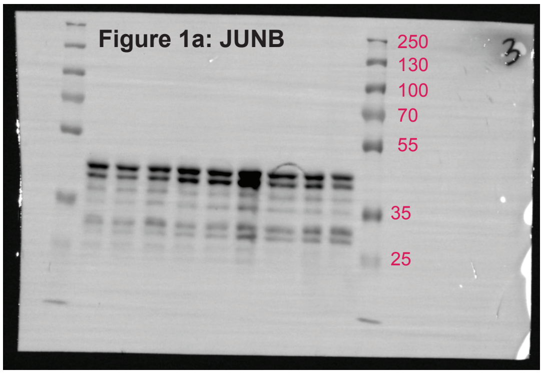

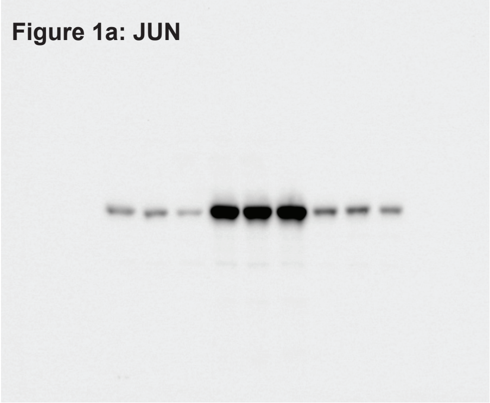

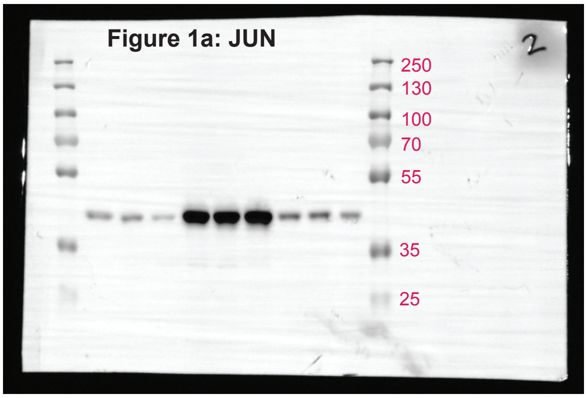

Figure S1. Uncropped Western blots. The chemiluminescence blot used to create Figure 1a is shown for each protein probed (left side). The colormetric image merged to show ladder marks is also included on the right side with ladder sizes marked in red for each blot. .

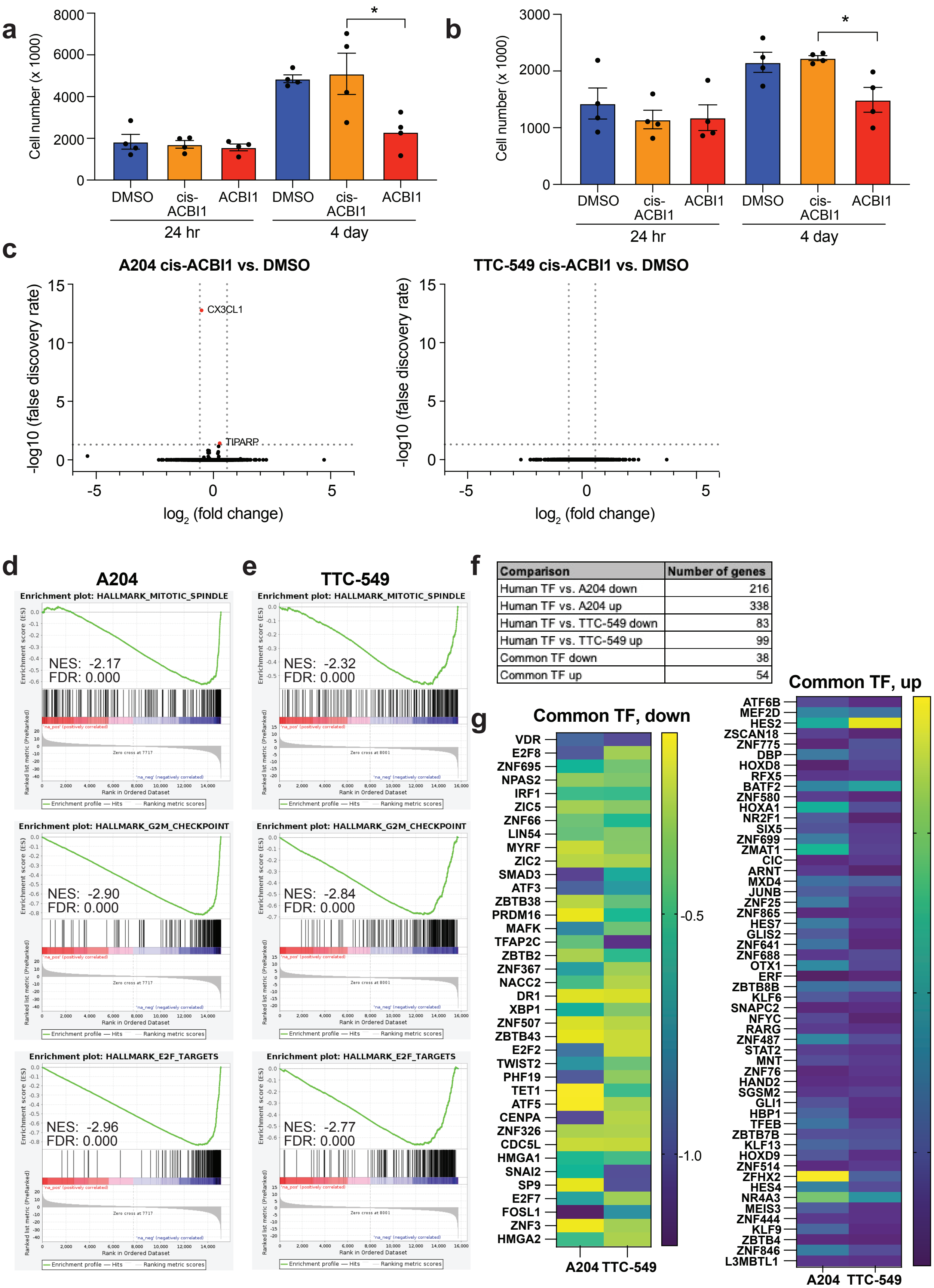

Comparison	Number of genes
Human TF vs. A204 down	216
Human TF vs. A204 up	338
Human TF vs. TTC-549 down	83
Human TF vs. TTC-549 up	99
Common TF down	38
Common TF up	54

Figure S2. Transcriptome analysis of diverse rhabdoid cell lines. (a) Equal numbers of A204 cells were plated with the indicated treatments. Total cell numbers for all treatments were determined 24 hr or 4 days later. Dots on each bar correspond to the data point obtained per replicate (n = 4 biological replicates, error bars are standard error of the mean, *P = 0.041 using unpaired t-test, two-tailed). (b) Equal numbers of TTC-549 cells were plated and counted similarly to A204 cells (n = 4 biological replicates, error bars are standard error of the mean, *P = 0.016 using unpaired t-test, two-tailed). (c) Volcano plot showing magnitude and significance of all genes detected in RNA-seq analysis between cis-ACBI1 vs. DMSO for A204 or TTC-549 cells. Red indicates significantly changed genes (FDR < 0.05). The two genes significantly changed in A204 cells are labelled with their gene name. Grey lines mark a FDR of 0.05 and fold change of 1.5. Gene set enrichment analysis was performed in A204 cells (d) or TTC-549 cells (e) using ACBI1 vs. cis-ACBI1 gene expression changes and the MSigDB hallmark data sets for each cell line. Genes were negatively enriched within the gene sets indicated, with normalized enrichment score (NES) and FDR included. (f) A published list of human transcription factor (TF) genes were compared to genes that decrease (down) or increase (up) in expression following ACBI1 treatment. Numbers of genes from each comparison are shown. The number of transcription factors that change expression in the same direction in both A204 and TTC-549 cells (i.e., common) are also listed. (g) Heatmap showing the log2-fold change in expression from the RNA-seq analysis for the common transcription factors determined in (f). Left heatmap shows the name of all transcription factors that were commonly decreased in expression compared to the right heatmap which shows those commonly increased.

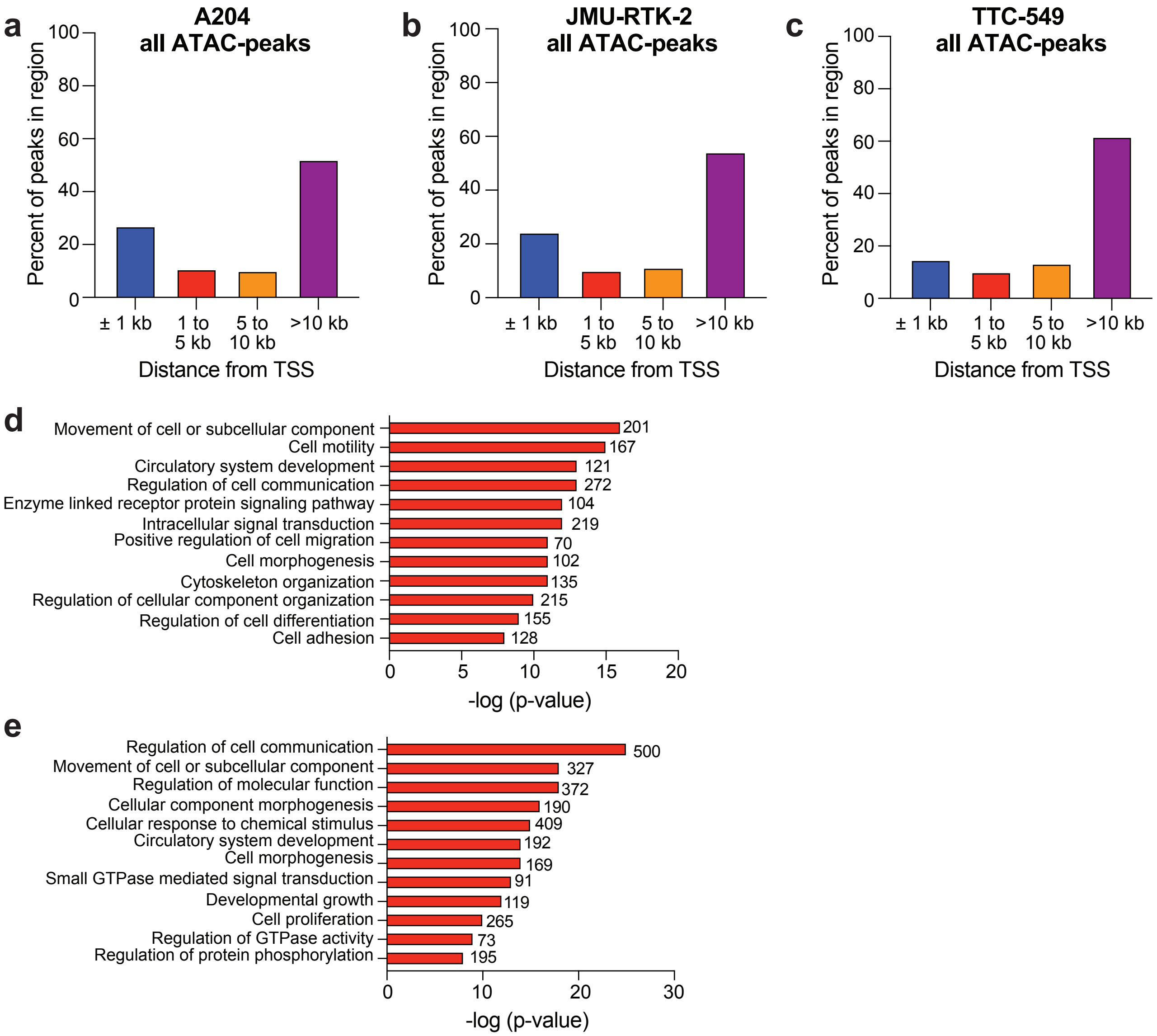

Figure S3. Additional ATAC-seq analysis of diverse rhabdoid cell lines. (a) All ATAC-peaks detected and included in differential analysis (for A204 cells) were annotated and distance of each peak to the nearest transcription start site (TSS) was determined. Bars show the percent of total peaks in each region. Similar analysis was performed for JMU-RTK-2 (b) and TTC-549 (c) cells. (d) GO-term analysis performed on BRG1-accessible sites in the JUM-RTK-2 cell line (FDR < 0.05). The number of genes in each category are noted next to the bar and significance of enrichment within each category is on the x-axis. Similar analysis was performed for TTC-549 cells (e) (FDR < 0.05, fold change < -1.5).

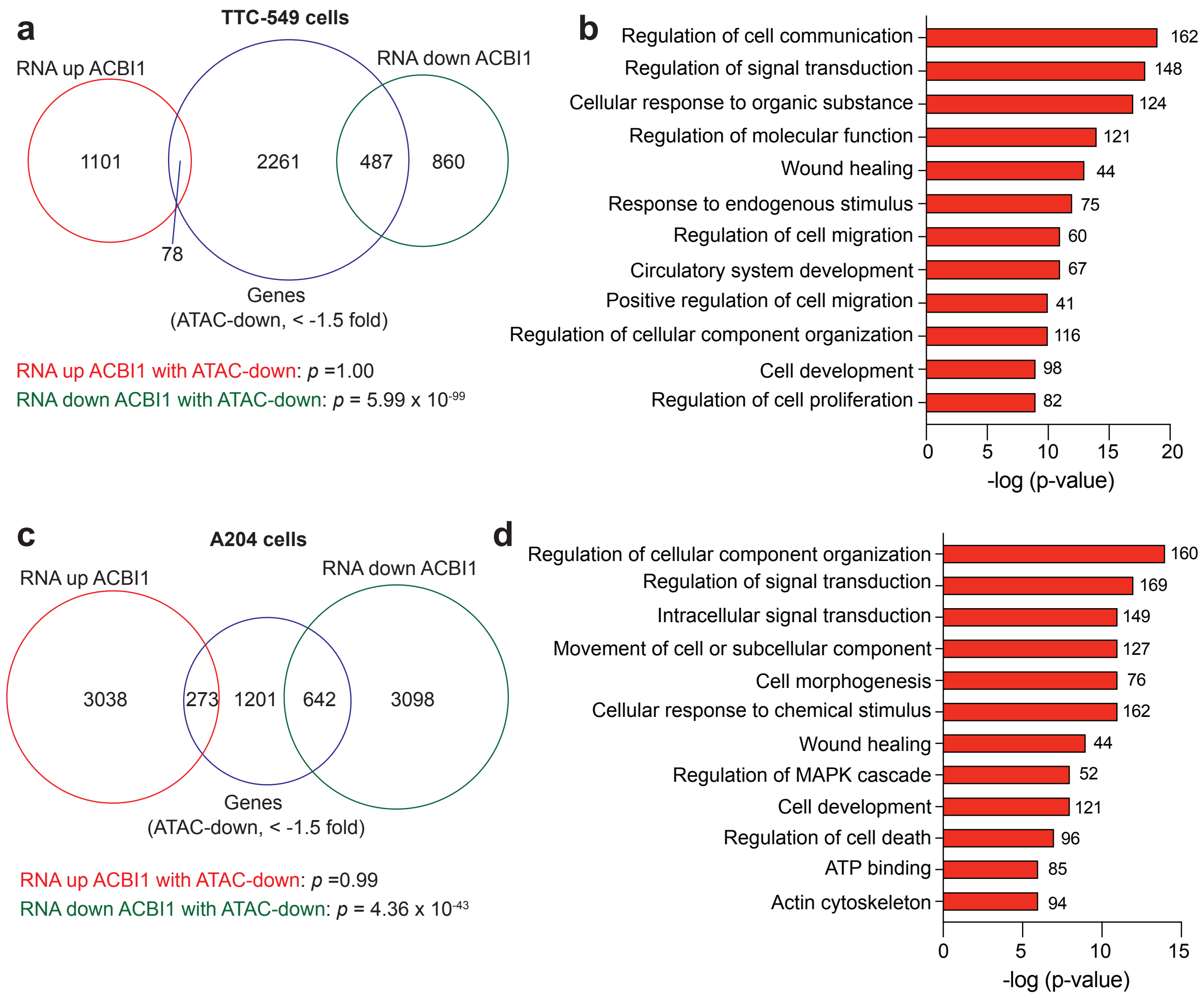

Figure S4. Comparison of RNA-seq and ATAC-seq analysis in TTC-549 and A204 cell lines. (a) Venn diagram showing the overlap of genes that were increased (up) or decreased (down) in expression compared to the genes annotated to ATAC-peaks that decreased in intensity (FDR < 0.05, Fold change < -1.5). Data are shown for TTC-549 cells and p-value resulting from the two gene overlaps is shown and was determined by performing a hypergeometric test. (b) GO-term analysis of 487 genes in (a). The number of genes in each category are noted next to the bar and significance of enrichment within each category is on the x-axis. (c) Venn diagram as shown in (a), except data are shown for A204 cells. (d) GO-term analysis of 642 genes in (c). The number of genes in each category are noted next to the bar and significance of enrichment within each category is on the x-axis.
